# Association of diet and lifestyle factors with semen quality in male partners of Chinese couples preparing for pregnancy

**DOI:** 10.1186/s12978-023-01718-5

**Published:** 2023-11-23

**Authors:** Hanran Mai, Junyi Ke, Zilin Zheng, Jieyi Luo, Miaomiao Li, Yanxia Qu, Fan Jiang, Simian Cai, Liandong Zuo

**Affiliations:** 1 Department of Clinical Biological Resource Bank, Guangzhou Institute of Pediatrics, Guangzhou Women and Children’s Medical Center, Guangzhou Medical University, Guangzhou, 510623 China; 2 Department of Andrology, Guangzhou Women and Children’s Medical Center, Guangzhou Medical University, 9 Jinsui Road, Guangzhou, 510623 Guangdong China; 3 Department of Gynecology, Guangzhou Women and Children’s Medical Center, Guangzhou Medical University, Guangzhou, 510623 China; 4 Prenatal Diagnostic Center, Guangzhou Women and Children’s Medical Center, Guangzhou, 510623 Guangdong China; 5 Department of Science, Education and Data Management, Guangzhou Women and Children’s Medical Center, Guangzhou, 510623 Guangdong China

**Keywords:** Semen quality, Male fertility, Diet factors, Lifestyle factors

## Abstract

**Background:**

Semen quality significantly influences conception, and its preservation is crucial for couples seeking pregnancy. We investigated dietary and lifestyle risk factors impacting semen quality.

**Methods:**

A total of 466 males from the Guangzhou Women and Children’s Medical Center’s pre-pregnancy consultation clinic were recruited between January 2021 and March 2023 for inclusion. Semen analysis was performed, and diet and lifestyle data were gathered via questionnaire. Logistic regression was utilized to examine the link between diet, lifestyle variables, and semen quality.

**Results:**

Smoking worsened progressive sperm motility (38.0% vs. 36.0%, t = 2.262; P = 0.049). Alcohol consumption impaired progressive motility (40.5 ± 17.8% vs. 34.7 ± 16.1%, t = 3.396; P < 0.001) and total motility (56.0% vs. 64.0%; P = 0.001). Using plastic beverage bottles for oil or seasonings lowered sperm concentrations (40.4% vs. 59.0% vs. 65.5%; P = 0.032). A sweet diet correlated with higher total sperm motility (55.0% vs. 60.0%, 62.0% vs. 63.2%; P = 0.017). Higher milk product intake improved sperm concentration (41.6*10*^6^ *vs. 63.7*10^6^ vs. 66.1*10^6^; P = 0.021) and motility (54.5% vs. 56.0% vs. 63.0%; P = 0.033). More frequent egg consumption increased semen volume (3.1 mL vs. 3.8 mL vs. 4.0 mL; P = 0.038). Roughage intake enhanced sperm concentration (160.8*10*^6^ vs.* 224.6*10^6^; P = 0.027), and adequate sleep improved progressive sperm motility rate (35.4% ± 18.2% vs. 40.2 ± 16.3%, F = 3.747; P = 0.024) and total motility (52.7% vs. 61.5%; P = 0.013). The regression model showed that using plastic containers for condiments was a protective factor for semen volume (OR: 0.12; CI 0.03–0.55; P = 0.006), sperm concentration (OR: 0.001, CI 0.00–0.30; P = 0.012), and count (OR: 0.12, CI 0.03–0.48; P = 0.003). Milk and egg consumption were also protective for semen volume (OR: 0.18, CI 0.06–0.51; P = 0.001 and OR: 0.11, CI 0.03–0.55; P = 0.006, respectively), while sufficient sleep benefitted total sperm motility (OR: 0.47, CI 0.24–0.95; P = 0.034).

**Conclusions:**

Smoking and drinking, type of condiment container, diet preference, sleep duration, and milk, roughage, and egg consumption may reduce semen quality.

## Background

Couples who fail to conceive after 12 months of regular unprotected sexual activity are defined as infertile. In China, infertility has risen from approximately 12% to 18% as a result of improvements in education levels and quality of life, as well as changes in the concept of childbearing, and delays in the age of marriage and pregnancy [1]. Studies have shown that semen quality in adult males has been declining globally and has stabilized at low levels in recent decades [2]. Although the underlying causes of declining semen quality are the focus of current research, poor dietary habits and lifestyles may go some way to explain these trends. Therefore, there is an urgent need to identify the risk factors associated with infertility to help couples to restore their fertility.

Infertility affects both males and females. According to Sengupta P et al., male factors cause infertility in up to 40% of couples [3]. In clinical and scientific work, semen parameters, including semen volume, sperm concentration, sperm count, sperm progressive motility, and total motility, are often used as indicators to assess male fertility [4]. Spermatogenesis is a dynamically changing physiological process [5], which is easily influenced by lifestyle and diet. A review by Ostojic pointed out that creatine is a potential supplement for couples preparing for pregnancy, while another review indicated that the intake of myo-inositol is an effective supplement for sperm quality [6]. The muscles of animals, such as beef and pork, are rich in creatine and myo-inositol, which are difficult to obtain. Herbal foodstuffs such as onions, garlic, and carrots contain numerous nutrients that have positive effects on testosterone production and improve semen quality. Overall, a varied and balanced diet is important for maintaining good semen quality [7].

Unhealthy lifestyle is another unavoidable aspect of semen quality. Indeed, there is a consensus about moderate exercise as a positive factor for semen quality [8–10]. This may be because men who undertake moderate exercise have higher metabolic levels and better body shape, which are protective against obesity—a negative factor for semen quality [11]. Moderate abstinence has also been reported to be a positive factor for semen volume, total motility, and sperm concerntration [12]. Additionally, the World Health Organization (WHO) advised that patients should have 3–7 days of abstinence before collecting samples for semen analysis [13]. Furthermore, a study from Ghana showed that sitting for a long time and smoking were both related to lower sperm count [14].

Lifestyle and dietary factors have been shown to impact semen quality. Nevertheless, given the myriad of potential influences in lifestyle and diet, numerous factors remain unexamined in their relation to semen quality. Also, given that the confounding factors are vast, relevant studies are still needed to clarify which factors are affecting the human sperm quality. To investigate unknown life forms and environmental determinants of semen quality, and partially validate other studies' findings, we developed three questionnaires focusing on demographic traits, dietary patterns, and lifestyle factor exposures, tailored to the living habits of the Chinese population.

Based on our fertility cohort, more than 466 couples were enrolled between June 2020 and July 2021. We collected the couples’ essential information and completed diet as well as lifestyle factor questionnaires to verify the effects of certain lifestyles and diet factor on semen, explore more lifestyle and dietary factors related to semen quality, and guide couples in healthy pregnancy preparation and promote sexual reproductive health.

## Methods

### Study population

We enrolled couples from the pre-pregnancy consultation clinic of the Guangzhou Women and Children’s Medical Center in Guangzhou, China, who were invited to participate in a prospective cohort study that focused on whether lifestyle and dietary factors influenced fertility. After normalizing the female partners’ confounding factors and excluding male partners who had a medical history of systemic diseases and infertility-related diseases (including varicocele, cryptorchidism, and azoospermia), a total of 466 couples were included in this study between January 2021 and March 2023. Male partners aged 30–42 years completed three questionnaires relating to lifestyle, diet, and demographic information. All of the couples were East Asian.

### Physical examination and semen analysis

Physical examinations and semen analysis were performed on the same day. The testicles and scrotum of each participant were examined to exclude patients with varicocele or other reproductive organ abnormalities.

The participants were required to abstain from sex for 3–7 days before semen analysis and physical examination. Semen samples were collected in a sterile semen container following masturbation and placed in a 37 °C incubator for 30 min to liquefy. After liquefaction, semen analysis was performed using computer-aided sperm analysis (CASA, SuiJia Software, Beijing, China) to evaluate the semen pH, volume, concentration, count, progressive motility, and total motility. All operations and reference values for semen parameters were in accordance with the latest guidelines of the WHO [13].

Our laboratory regularly conducts quality control screening to ensure the quality of semen analysis results.

### Diet and lifestyle questionnaires

Based on the living habits of people in China, we designed two individual questionnaires to assess participants’ diet and lifestyle exposures. We also designed elaborate questions for factors related to low semen quality, such as smoking [15, 16], alcohol consumption [17, 18], and duration of sleep [19, 20]. We also designed additional questions based on the participants’ demographic characteristics. All of the study questionnaires used choice questions.

### Ethics statement

The study was approved by the Ethics Review Committee of Guangzhou Women and Children’s Medical Center. Written informed consent was obtained from all of the participants.

### Statistical analysis

The Shapiro–Wilk test was used to assess the normality of the data. None of the semen quality parameters were normal except for progressive motility (%). Normally distributed data are presented as the mean ± standard deviation, whereas other data are presented as the median (25th and 75th percentiles). Associations among semen quality parameters, diet, and lifestyle factors were evaluated. The Mann–Whitney U-test and Kruskal–Wallis H test were used for data with a non-normal distribution, and ANOVA was used for normally distributed data.

To further explore the association between semen quality and environmental and occupational factors, binomial logistic regression was applied to detect independent predictors that significantly affected semen quality, and the following confounders were adjusted for in the analysis: education [21], BMI [22] and age [23]. Statistical significance was set at P < 0.05. Statistical analyses were performed using SPSS version 26.0 (SPSS Inc., Chicago, IL, USA).

## Result

### Characteristics of the study population

As shown in Table 1, we enrolled 466 males of reproductive age, with a mean age of 37.53 ± 5.75 years. Every participant had a stable job and was willing to accept follow-up services. Our study included individuals with varying degrees of education.

**Table 1:** General characteristics of the study population (n = 466)

Variables	N (%) or mean ± SD
Age, years	36.53 ± 5.75
Nationality, n (%)	
Han nationality	456 (97.94)
Other	10 (2.06)
Education, n (%)	
Primary school and below	13 (2.72)
Junior high school	67 (14.36)
High school	165 (35.40)
College or university degree	197 (42.33)
A master’s degree	22 (4.70)
PhD	2 (0.50)

### Semen quality

The results showed that the median (25th, 75th percentiles) semen pH, volume, concentration, count, and total motility were 7.3 (7.2–7.5), 3.6 (2.5–5.0) mL, 63.6 (38.3–100.0) * 10^6^**/**mL, 213.7 (121.8–422.0) * 10^6^/mL, and 58.0 (42.0–73.0)%, respectively. Additionally, the mean ± SD progressive sperm motility was 36.4% ± 16.8% (Table 2).

**Table 2:** Summary of semen parameters of males

Variables	Statistics
Semen volume(ml), Median (25th, 75th percentiles)	3.6 (2.5–5.0)
Sperm progressive motility (%), Mean ± SD	36.0 (23.0–49.0)
Total motility (%), Median (25th, 75th percentiles)	58.0 (42.0–73.0)
Sperm concentration (10^6^ mL^−1^), Median (25th, 75th percentiles)	63.6 (38.3–100.0)
Sperm count (10^6^ mL^−1^), Median (25th, 75th percentiles)	213.7 (121.8–422.0)
pH value, Median (25th, 75th percentiles)	7.3 (7.2–7.5)

### Association between diet and lifestyle factors with semen quality

As mentioned above, none of the semen parameters fit a normal distribution except for progressive motility (%). The Mann–Whitney U-test and Kruskal–Wallis H test were applied for all skewness distribution semen parameter data analyses. Analysis of variance (ANOVA) was applied to analyze normally distributed semen measurements. Our results suggested that smoking (38.0% vs. 36.0%, for no and yes, respectively; t = 2.262; P = 0.049) and alcohol consumption (64.0% vs. 56.0% for no and yes, respectively; P = 0.001) decreased the progressive sperm motility, while alcohol consumption significantly decreases the total sperm motility (40.5% ± 17.8% vs. 34.7% ± 16.1% for no and yes, respectively; t = 3.396; P < 0.001). The frequency of using plastic beverage bottles as containers for cooking oil and condiments was a negative factor for sperm concentration (65.5 * 10^6^/mL vs. 59.0 * 10^6^/mL vs. 40.4 * 10^6^/mL for never, occasionally, and often, respectively; P = 0.032). According to our results, taste preference was also related to total sperm motility (55.0% vs. 63.2% vs. 62.0% vs. 60.0% for partial light, partial sweet, partial salty, and partial greasy, respectively; P = 0.017). Moreover, the frequency of eating roughage was a positive factor for the total sperm count (224.6 * 10^6^ vs. 160.8 * 10^6^ for occasionally and basically do not; P = 0.042), while the frequency of consuming milk and dairy products was beneficial to the total sperm motility (63.0% vs. 56.0% vs. 54.5% for every day, occasionally, and basically do not, respectively; P = 0.021) and sperm concentration (66.1 * 10^6^/mL vs. 63.7 * 10^6^/mL vs. 41.6 * 10^6^/mL for every day, occasionally, and basically do not, respectively; P = 0.033). Our results suggested that eating eggs constantly may contribute to increased semen volume (4.0 mL vs. 3.8 mL vs. 3.1 mL for every day, occasionally, and basically do not, respectively). Moreover, sufficient sleep was vital to total sperm motility (61.5% vs. 57.0% vs. 52.7% for do not feel sleepy, feel sleepy occasionally, and often feel sleepy; P = 0.013) and progressive sperm motility (40.2% ± 16.3% vs. 35.2% ± 16.5% vs. 35.4% ± 18.2% for do not feel sleepy, feel sleepy occasionally, and often feel sleepy; F = 3.747; P = 0.024). We also found that several factors significantly affected the semen pH value, but there was no significant change in the pH value, The difference in significance and the statistical analyses that were conducted to obtain these results are unclear. According to the WHO guidelines, a pH value > 7.2 and < 7.8 is normal for a healthy man, and none of the study participants showed abnormal pH values [13]. Therefore, further research is needed to determine whether lifestyle and dietary factors can affect semen pH (Tables 3 and 4).

**Table 3:** Description of semen parameters in different dietary intake

Characteristic	N	Semen volume(ml)		Progressive motility (%)		Total motility (%)		Sperm concentration(10^6^/ml)		Sperm count (10^6^/ml)		pH value	
		Median (25th, 75th)	Effect size	Median (25th, 75th)	Effect size	Median (25th, 75th)	Effect size	Median (25th, 75th)	Effect size	Median (25th, 75th)	Effect size	Median (25th, 75th)	Effect size
Drinking													
Yes	135	3.6 (2.5–5.0)	− 0.016	40.0 (25.0–54.0)	0.347	56.0 (40.0–71.0)	0.379	61.8 (37.8–98.7)	0.057	209.5 (121.9–389.6)	0.061	7.3 (7.2–7.5)	0.039
No	327	3.5 (2.6–5.0)		33.5 (18.3–48.8)^*^		64.0 (49.0–79.0)^*^		68.0 (41.3–104.8)		246.0 (119.3–477.4)		7.3 (7.2–7.5)	
Frequency of alcohol consumption													
Often	14	5.0 (4.2–6.1)	0.446	38.5 (29.8–54.3)	− 0.021	66.5 (36.5–77.9)	− 0.131	70.2 (40.2–105.5)	− 0.128	355.0 (148.1–580.7)	0.155	7.4 (7.2–7.6)	0.081
Occasionally	126	3.4 (2.6–5.0)		41.0 (25.0–55.5)		62.9 (49.0–79.0)		68.5 (40.7–110.3)		242.4 (113.9–489.7)		7.2 (7.2–7.5)	
Never	68	3.6 (2.5–6.0)		33.0 (18.5–47.5)		60.0 (40.2–74.8)		65.1 (50.7–105.5)		261.0 (159.8–472.6)		7.3 (7.2–7.5)	
Less	8	2.2 (1.9–4.5)		30.0 (17.5–45.3)		45.4 (35.5–64.1)		55.4 (27.1–77.5)		153.3 (46.2–285.4)		7.2 (7.1–7.5)	
Types of regular drinking													
Beer	51	4.1 (3.2–5.6)	0.026	43.0 (25.8–57.0)	0.017	67.0 (51.0–78.0)	0.023	73.6 (41.3–96.9)	0.014	294.4 (118.6–572.4)	0.014	7.2 (7.2–7.5)	0.003
Liquor	27	4.2 (3.2–6.0)^*^		41.0 (25.0–48.0)		61.2 (52.0–74.4)		72.9 (35.9–151.3)		305.7 (160.8–560.2)		7.2 (7.2–7.5)	
Wine	31	3.1 (2.2–4.0)		38.0 (21.0–53.0)		55.0 (37.0–72.0)		66.6 (42.3–128.0)		189.6 (96.1–433.1)		7.2 (7.2–7.5)	
Miscellaneous	34	3.1 (2.2–4.7)		37.0 (23.5–58.3)		67.5 (48.6–86.1)		72.6 (42.8–117.1)		221.7 (128.1–418.5)		7.3 (7.2–7.5)	
Other	50	3.6 (2.5–6.1)		34.0 (19.0–47.0)		56.9 (33.8–71.0)		63.8 (38.6–104.8)		264.2 (114.4–523.1)		7.3 (7.2–7.5)	
The consumption of alcohol everyday													
Less than 100 mL per day	152	3.5 (2.5–5.3)	0.045	38.0 (24.0–52.0)	0.025	61.0 (47.8–74.0)	0.049	66.7 (41.5–108.8)	0.016	259.5 (119.3–500.8)	0.035	7.3 (7.2–7.5)	0.007
100–250 mL per day	12	4.8 (2.6–6.0)		39.5 (24.5–54.8)		64.0 (38.8–81.6)		82.2 (49.6–128.9)		372.5 (136.5–544.0)		7.3 (7.2–7.5)	
More than 250 mL per day	10	4.8 (2.8–6.3)		45.5 (29.5–58.5)		67.0 (43.3–84.4)		50.1 (39.2–95.2)		172.7 (123.2–616.3)		7.4 (7.2–7.6)	
0 mL per day	6	3.5 (2.3–4.9)		38.5 (15.0–56.5)		53.5 (34.7–82.5)		72.6 (40.2–113.7)		253.4 (133.4–500.5)		7.2 (7.2–7.5)	
Daily water intake													
Below 500 mL	55	3.5 (2.5–4.6)	0.009	44.0 (28.3–56.8)	0.008	66.0 (39.5–79.0)	0.003	71.6 (42.6–129.0)	0.005	245.2 (132.2–522.4)	0.002	7.2 (7.2–7.5)	0.001
500–2500 mL	360	3.6 (2.5–5.0)		38.0 (24.0–52.0)		58.0 (42.9–72.4)		62.5 (38.0–98.0)		209.1 (111.9–400.9)		7.3 (7.2–7.5)	
More than 2500 mL	47	4.2 (2.8–6.0)		35.5 (19.0–52.0)		55.0 (35.0–68.0)		62.1 (39.1–96.6)		211.2 (133.1–484.7)		7.3 (7.2–7.7)	
Types of drinking water for outdoor activities													
Mineral water	219	3.6 (2.5–5.0)	0.008	42.0 (25.0–53.0)	0.002	59.0 (42.0–72.9)	0.010	70.4 (41.5–98.2)	0.008	219.3 (129.1–414.5)	0.005	7.3 (7.2–7.5)	0.001
Pure water	77	3.5 (2.8–5.7)		40.0 (20.0–53.0)		56.0 (41.5–73.1)		55.5 (27.3–102.1)		203.8 (105.6–413.0)		7.2 (7.2–7.5)	
Tap water (boiled water)	109	3.7 (2.5–5.4)		38.5 (22.8–48.0)		56.0 (42.1–72.0)		59.0 (37.5–106.0)		222.2 (112.3–419.9)		7.3 (7.2–7.5)	
Tea	20	4.0 (2.2–6.0)		41.0 (23.0–56.0)		62.5 (41.4–74.5)		96.9 (40.8–145.0)		307.9 (142.6–578.6)		7.3 (7.2–7.5)	
Beverage	37	3.6 (2.8–4.8)		36.0 (20.5–45.0)		60.0 (40.7–75.0)		51.8 (38.2–86.1)		181.7 (130.8–424.5)		7.3 (7.2–7.5)	
Beverages that often consumed													
Pure juice	37	3.5 (2.3–5.6)	0.005	44.0 (31.0–56.0)	0.001	64.1 (44.0–79.5)	0.001	65.8 (39.2–96.8)	− 0.002	202.5 (130.0–517.9)	0.005	7.5 (7.2–7.6)	0.006
Non-carbonated sugar-sweetened beverages	37	3.8 (3.1–4.3)		37.0 (26.8–42.3)		50.0 (35.5–66.7)		52.4 (36.6–97.6)		181.7 (135.5–347.5)		7.3 (7.2–7.5)	
Coffee	18	3.4 (2.6–4.2)		39.5 (29.5–55.8)		59.1 (46.6–69.7)		65.6 (27.7–102.9)		240.7 (96.1–397.5)		7.3 (7.2–7.7)	
Carbonated drinks	89	4.0 (3.0–5.6)		36.0 (22.0–54.8)		61.0 (43.5–75.5)		67.4 (33.4–93.3)		240.2 (132.7–541.3)		7.3 (7.2–7.5)	
Hardly drink	281	3.5 (2.5–5.0)		40.0 (24.0–52.0)		57.9 (41.5–72.3)		61.8 (39.9–106.7)		213.9 (115.1–415.7)		7.2 (7.2–7.5)	
Number of drinks purchased in a week													
≤ 3 bottles	407	3.5 (2.5–5.0)	0.011	39.5 (23.8–52.3)	0.012	58.0 (42.0–73.0)	0.012	63.7 (38.3–99.9)	0.003	213.9 (120.4–416.9)	0.005	7.3 (7.2–7.5)	0.009
4–6 bottles	43	4.0 (3.2–5.0)		40.0 (24.8–54.0)		58.0 (44.0–75.0)		64.2 (39.8–114.0)		233.6 (143.5–569.9)		7.3 (7.2–7.5)	
7–9 bottles	6	3.8 (1.4–4.7)		29.5 (22.0-)		61.0 (33.4–70.5)		49.1 (25.0–79.7)		120.2 (72.0–318.7)		7.5 (7.2–7.5)	
≥ 10 bottles	6	3.3 (2.6–4.7)		32.0 (24.5–48.0)		45.0 (29.5–67.2)		56.0 (37.9–140.5)		209.1 (122.4–418.1)		7.4 (7.2–7.7)	
Use plastic beverage bottles for cooking oil, condiments, etc													
Never	301	3.5 (2.5–5.0)	0.004	40.0 (25.0–53.0)	0.004	59.0 (43.5–72.3)	0.007	65.5 (41.4–104.7)^*^	0.010	222.2 (129.6–421.2)	0.004	7.2 (7.2–7.5)	0.001
Occasionally	149	3.8 (2.5–5.3)		38.5 (21.5–52.3)		56.0 (42.0–74.1)		59.0 (33.4–96.1)		203.8 (106.8–430.3)		7.3 (7.2–7.5)	
Often	12	3.2 (2.1–6.1)		34.5 (10.3–42.3)		38.7 (34.4–56.3)		40.4 (21.9–73.2)		130.7 (50.8–403.8)		7.4 (7.2–7.7)	
Dietary preference													
Light	270	3.8 (2.5–5.0)	0.008	37.0 (24.0–52.3)	0.012	55.0 (39.0–71.0)	0.021	61.6 (36.6–102.0)	0.004	224.6 (116.3–403.0)	0.002	7.3 (7.2–7.5)	0.007
Partial sweet	38	3.8 (2.5–5.5)		33.0 (22.3–51.3)		63.2 (42.0–72.4)^*^		63.2 (37.0–135.3)		280.9 (130.8–591.0)		7.2 (7.2–7.5)	
Partial salty	107	3.6 (2.7–5.0)		44.0 (26.0–54.0)		62.0 (47.8–77.0)		66.1 (42.6–96.6)		211.7 (129.5–429.4)		7.2 (7.2–7.5)	
Partial greasy	47	3.2 (2.4–4.6)		40.5 (28.3–53.0)		60.0 (51.0–79.0)		57.5 (35.9–88.1)		179.3 (97.9–442.4)		7.3 (7.2–7.7)	
Eat pickled foods													
Never	25	4.0 (2.4–6.2)	0.060	36.0 (20.3–53.0)	0.001	61.0 (42.6–74.0)	0.001	65.1 (46.1–87.8)	0.000	249.2 (149.8–386.1)	0.006	7.2 (7.2–7.5)	0.004
Occasionally	421	3.6 (2.5–5.0)		39.0 (24.0–52.0)		58.0 (42.0–72.3)		63.7 (38.6–99.9)		218.4 (120.5–425.1)		7.3 (7.2–7.5)	
Often	15	3.0 (2.0–4.0)		32.0 (20.0–67.0)		50.0 (21.0–87.1)		58.2 (27.2–104.0)		163.0 (90.3–332.8)		7.2 (7.2–7.5)	
Almost every day	1	–		–		–		–		–		–	
Eat fried food													
Never	18	4.7 (2.8–6.4)	0.009	51.0 (21.3–56.8)	0.000	67.5 (38.5–79.7)	0.001	74.1 (50.3–130.4)	0.001	328.6 (181.7–432.1)	0.003	7.2 (7.2–7.3)	0.012
Occasionally	430	3.5 (2.5–5.0)		39.0 (24.0–52.0)		58.0 (42.0–72.2)		62.3 (37.8–99.7)		205.5 (119.3–417.5)		7.3 (7.2–7.5)	
Often	14	4.5 (2.2–6.7)		39.5 (34.0–48.3)		55.2 (50.3–70.1)		73.7 (44.2–96.9)		314.7 (121.8–487.9)		7.5 (7.2–7.7)	
Daily meal plan													
Mainly meat	48	3.5 (2.7–5.9)	0.000	51.0 (21.3–56.8)	0.007	61.5 (46.9–80.9)	0.011	71.7 (44.6–118.4)	0.005	295.9 (126.0–610.3)	0.006	7.2 (7.2–7.5)	0.022
Half meat and half vegetable	378	3.6 (2.5–5.0)		39.0 (24.0–52.0)		58.0 (42.0–72.0)		62.4 (37.9–99.0)		211.5 (120.1–397.2)		7.3 (7.2–7.5)	
Mainly vegetarian	36	4.3 (2.5–5.3)		39.5 (34.0–48.3)		54.7 (38.1–69.3)		61.0 (41.6–91.2)		221.5 (128.3–399.0)		7.2 (7.2–7.3)	
Frequency of eating whole grains													
Occasionally	421	3.6 (2.5–5.0)	0.247	39.5 (23.3–52.8)	0.019	58.0 (42.0–73.0)	0.003	65.0 (39.1–101.0)	0.203	224.6 (125.9–425.1)	0.176	7.3 (7.2–7.5)	0.061
Basically don’t	41	3.4 (2.6–5.0)		39.0 (25.0–50.0)		55.0 (40.1–73.1)		50.3 (33.3–90.1)		160.8 (97.9–361.1)		7.2 (7.2–7.5)	
Frequency of eating soy products													
Every day	16	3.5 (2.2–5.0)	0.002	38.5 (17.5–56.3)	0.000	51.1 (42.2–80.7)	0.000	51.6 (23.3–103.4)	0.003	170.6 (97.6–263.9)	0.003	7.3 (7.2–7.7)	0.000
Occasionally	430	3.6 (2.5–5.0)		39.5 (24.0–52.0)		58.6 (42.0–73.0)		62.8 (38.3–98.6)		214.4 (123.8–423.0)		7.3 (7.2–7.5)	
Basically don't	16	3.2 (2.1–5.0)		25.0 (24.0-)		52.4 (42.7–71.0)		104.2 (61.5–127.3)		366.1 (119.2–525.3)		7.2 (7.2–7.7)	
Consumption of milk and dairy products													
Every day	89	3.4 (2.5–5.0)	0.003	41.0 (21.3–55.3)	0.005	63.0 (48.9–78.0)^*^	0.017	66.1 (45.7–103.2)^*^	0.009	218.4 (145.9–378.2)	0.009	7.2 (7.2–7.5)	0.008
Occasionally	351	3.8 (2.5–5.0)		38.0 (24.0–50.3)		56.0 (41.0–71.0)		63.7 (37.9–100.8)		224.5 (119.3–433.1)		7.3 (7.2–7.5)	
Basically don’t	22	4.0 (2.1–5.1)		44.0 (20.0–59.5)		54.5 (31.8–81.5)		41.6 (27.9–67.0)		126.3 (78.5–316.0)		7.3 (7.2–7.5)	
Frequency of eating eggs													
Every day	86	4.0 (3.1–5.0)^*^	0.014	42.0 (24.0–56.0)	0.010	56.5 (42.5–74.4)	0.007	61.5 (40.7–108.9)	0.002	189.3 (100.8–342.4)	0.005	7.2 (7.2–7.5)	0.005
Occasionally	369	3.8 (2.7–5.0)		39.0 (24.3–52.0)		58.9 (42.0–73.0)		64.0 (38.3–99.2)		228.9 (126.2–432.1)		7.3 (7.2–7.5)	
Basically don't	7	3.1 (2.1–5.0)		20.0 (7.0–41.3)		39.0 (36.0–55.6)		42.2 (24.3–97.2)		211.2 (61.9–388.9)		7.3 (7.2–7.5)	
Which part of the egg do you eat													
Whole egg	408	3.5 (2.5–5.0)	0.004	38.5 (23.8–52.0)	0.008	57.9 (42.0–72.4)	0.012	61.5 (38.2–98.4)	0.008	205.5 (119.9–402.9)	0.012	7.3 (7.2–7.5)	0.003
Remove the yolks	52	4.1 (3.3–5.9)		40.0 (27.0–52.0)		64.2 (43.0–73.4)		81.9 (40.7–112.5)		296.8 (127.9–527.1)		7.3 (7.2–7.5)	
Basically don’t	2	4.1 (4.0-.)		57.5 (57.0-)		90.5 (88.0-.)		107.6 (84.6-.)		434.7 (346.9-.)			
Frequency of eating animal food (pork, beef, lamb)													
Every day	278	3.5 (2.5–5.0)	0.006	40.0 (24.0–53.0)	0.002	58.0 (42.0–73.9)	0.001	59.9 (35.0–99.0)	0.005	201.9 (106.3–388.1)	0.009	7.2 (7.2–7.5)	0.005
Occasionally	180	3.8 (2.5–5.3)		38.0 (22.3–52.0)		59.0 (42.5–72.0)		66.8 (41.4–106.8)		240.0 (131.6–452.5)		7.3 (7.2–7.5)	
Basically don’t	4	5.1 (2.8–8.8)		40.3 (22.4–52.0)		51.5 (46.3–56.8)		48.9 (42.5–58.2)		229.8 (142.2–420.1)		7.3 (7.2–7.5)	
Vegetarian													
Yes, but there is a corresponding daily intake of egg and milk	200	3.8 (2.6–5.1)	0.003	41.0 (22.3–52.0)	0.001	56.9 (44.0–72.0)	0.007	64.1 (39.0–102.1)	0.006	217.1 (123.2–392.8)	0.004	7.3 (7.2–7.5)	0.008
No, I like to eat meat, especially fatty meat	248	3.5 (2.5–5.0)		38.0 (25.0–54.0)		59.0 (42.0–74.8)		61.1 (38.0–97.1)		210.4 (115.3–454.7)		7.3 (7.2–7.5)	
Yes, completely vegetarian	14	3.7 (2.3–5.6)		39.5 (23.0–54.3)		54.5 (34.8–62.0)		102.9 (63.6–129.7)		375.7 (180.2–536.2)		7.2 (7.2–7.3)	
Frequency of eating animal viscera (liver, kidney, stomach, intestine)													
Basically don’t	64	3.6 (2.4–5.5)	0.113	38.0 (28.5–51.0)	− 0.001	57.8 (42.9–73.9)	− 0.143	63.2 (43.2–100.9)	0.013	218.5 (131.1–418.1)	0.096	7.3 (7.2–7.6)	0.042
Occasionally	398	3.6 (2.5–5.0)		40.0 (23.0–53.0)		58.0 (42.0–72.9)		63.6 (37.9–99.8)		212.8 (120.1–423.3)		7.3 (7.2–7.5)	
Frequency of eating dark colored vegetables such as yellow, red and purple													
Often	184	4.0 (2.5–5.0)	0.003	40.0 (23.0–53.0)	0.001	58.0 (43.7–73.0)	0.002	66.8 (40.9–107.7)	0.012	245.6 (133.1–459.5)^*^	0.016	7.3 (7.2–7.5)	0.002
Occasionally	274	3.5 (2.5–5.0)		39.0 (24.0–52.0)		58.3 (40.4–72.9)		57.8 (36.5–92.9)		186.3 (101.0–367.6)		7.3 (7.2–7.5)	
Basically don’t	4	2.6 (2.2–8.2)		39.0 (22.1–52.0)		46.0 (34.5–70.3)		105.7 (38.5–184.1)		243.8 (98.6–1546.4)		7.1 (6.9–7.8)	

The value of semen volume, progressive motility, sperm concentration, sperm count, total motility, pH value represent median (25th, 75th percentiles). *P < 0.05

**Table 4:** Description of semen parameters in different lifestyle

Characteristic	N	Semen volume(ml)		Progressive motility (%)		Total motility (%)		Sperm concentration(10^6^/ml)		Sperm count (10^6^/ml)		pH value	
		Median (25th, 75th)	Effect size	Median (25th, 75th)	Effect size	Median (25th, 75th)	Effect size	Median (25th, 75th)	Effect size	Median (25th, 75th)	Effect size	Median (25th, 75th)	Effect size
Smoking													
Yes	47	3.6 (2.5–5.0)	0.080	36.0 (22.0–48.0)	− 0.271	57.0 (42.0–72.0)	− 0.37	62.4 (38.3–99.8)	− 0.069	213.9 (121.6–408.1)	− 0.06	7.3 (7.2–7.5)	0.119
No	415	3.9 (3.0–4.6)		37.0 (27.0–57.3)^*^		67.0 (49.5–79.0)		72.9 (41.3–111.7)		236.3 (131.0–484.2)		7.2 (7.2–7.5)	
Losing weight or want to lose weight													
No need to lose weight	252	3.5 (2.5–5.0)	0.002	26.5 (19.3–48.0)	0.000	57.0 (42.0–72.0)	0.000	61.0 (38.0–98.1)	0.003	202.0 (118.8–388.6)	0.003	7.3 (7.2–7.5)	0.000
Want to lose weight	168	3.8 (2.7–5.2)		38.0 (25.0–48.5)		58.8 (41.4–73.4)		66.4 (36.4–107.1)		243.9 (130.8–473.0)		7.2 (7.2–7.5)	
Losing weight	42	3.6 (2.4–5.5)		36.0 (23.0–52.0)		58.0 (42.2–72.9)		62.7 (43.5–105.9)		224.6 (140.2–473.9)		7.3 (7.2–7.6)	
The way to lose weight													
Go on a diet	68	3.7 (2.7–5.0)	0.048	32.5 (20.3–46.0)	0.009	55.0 (37.5–69.1)	0.015	60.8 (34.2–87.7)	0.016	197.3 (130.8–344.9)	0.017	7.2 (7.2–7.5)	0.028
Sports	131	3.5 (2.4–5.0)		38.0 (23.3–50.8)		58.9 (42.2–74.2)		64.2 (36.5–107.4)		228.5 (110.7–481.3)		7.3 (7.2–7.5)	
Take diet pills	2	4.4 (3.5-.)		49.5 (47.0-)		71.5 (71.0-.)		54.6 (42.4-.)		229.1 (224.5-.)		7.4 (7.2-.)	
Eat weight loss health products (weight loss tea, meal replacement powder, etc.)	8	3.6 (1.1–4.6)		37.0 (22.3–47.8)		51.5 (41.5–68.5)		56.2 (39.7–86.1)		132.7 (77.6–333.2)		7.2 (7.2–7.6)	
Weight loss device	2	4.4 (2.7-.)		35.0 (21.0-)		64.0 (48.0-.)		106.5 (100.0-.)		473.9 (270.1-.)		7.7 (7.6-.)	
Other	41	4.6 (3.0–6.5)		38.0 (22.8–44.8)		57.0 (41.5–72.1)		64.2 (35.9–110.0)		332.4 (142.8–658.7)		7.5 (7.2–7.6)	
Duration of weight loss													
Less than 3 months	200	3.7 (2.5–5.3)	0.005	36.0 (22.0–49.0)	0.005	58.0 (41.0–72.4)	0.002	64.6 (39.0–97.1)	0.012	235.5 (129.7–464.4)	0.007	7.2 (7.2–7.5)	0.009
3–6 months	24	3.5 (2.0–4.6)		42.5 (25.0–52.8)		60.0 (37.1–79.8)		62.6 (34.6–108.4)		166.3 (133.7–334.2)		7.4 (7.2–7.5)	
6–12 months	5	3.6 (2.7–7.6)		41.0 (17.0–41.5)		58.0 (37.1–66.7)		35.2 (25.6–46.1)		149.6 (80.4–289.0)		7.5 (7.0–7.8)	
More than 12 months	17	3.8 (2.4–5.6)		30.0 (25.0–51.5)		54.0 (46.6–70.5)		97.9 (32.3–139.1)		270.1 (76.8–681.2)		7.5 (7.2–7.7)	
The average amount of sleep per day													
More than 8 h	49	4.0 (3.0–5.2)	0.000	36.0 (28.0–52.0)	0.002	59.0 (41.5–72.0)	0.001	65.5 (44.4–122.5)	0.014	272.5 (147.5–523.7)	0.017	7.3 (7.2–7.5)	0.002
Less than 6 h	22	3.6 (3.0–5.3)		36.0 (26.5–48.5)		58.7 (34.8–75.0)		42.5 (24.7–82.5)		159.9 (104.7–252.9)		7.3 (7.2–7.5)	
6–8 h	391	3.5 (2.5–5.0)		36.0 (21.5–49.0)		58.0 (42.0–73.0)		63.9 (38.3–99.7)		211.7 (121.9–423.5)		7.3 (7.2–7.5)	
The average time spent on phones in bed before going to bed each day													
Under half an hour	161	3.7 (2.5–5.4)	0.011	34.0 (20.5–48.0)	0.009	58.0 (40.0–73.3)	0.012	58.3 (39.9–98.5)	0.010	211.2 (121.3–389.4)	0.006	7.2 (7.2–7.5)	0.017
Half an hour to an hour	191	3.4 (2.5–5.0)		40.5 (25.0–52.0)		59.5 (45.1–75.0)		69.7 (41.3–108.2)		224.6 (125.4–433.0)		7.3 (7.2–7.5)	
1–2 h	80	4.0 (2.6–6.0)		33.5 (21.3–46.5)		58.0 (42.5–69.0)		65.4 (36.5–99.7)		262.7 (127.3–503.3)		7.4 (7.2–7.6)	
More than 2 h	30	3.7 (3.1–4.5)		34.0 (22.0–42.0)		52.0 (35.8–69.3)		43.8 (27.7–67.6)		158.2 (87.3–329.5)		7.4 (7.2–7.7)^*^	
Sleep time at night													
Before 22:00	39	4.6 (2.8–6.3)	0.016	42.5 (27.0–55.0)	0.018	63.0 (45.0–71.0)	0.014	81.0 (36.9–108.2)	0.021	355.2 (129.5–560.2)	0.027	7.4 (7.2–7.5)	0.003
22:00 to 24:00	320	3.6 (2.5–5.0)		33.0 (20.3–45.8)		56.5 (41.4–72.0)		62.0 (40.6–93.7)		213.7 (121.8–400.9)		7.3 (7.2–7.5)	
After 24:00	100	3.4 (2.5–5.0)		42.0 (28.0–52.5)		59.5 (44.2–80.0)		65.2 (34.3–111.2)		209.1 (118.5–430.6)		7.2 (7.2–7.5)	
All night long	3	5.0 (3.8-.)		42.5 (28.0–55.0)		35.0 (25.0-.)		28.5 (24.4-.)		146.3 (108.1-.)		7.5 (7.3-.)	
Difficulty falling asleep (can not fall asleep within 30 min)													
No	168	3.5 (2.3–5.0)	0.005	39.0 (22.0–52.0)	0.005	61.1 (44.2–75.3)	0.010	60.7 (37.8–95.7)	0.007	191.6 (112.2–367.6)	0.012	7.3 (7.2–7.5)^*^	0.024
Occasionally	241	3.8 (2.7–5.0)		35.5 (24.3–47.0)		56.0 (42.0–71.0)		63.5 (38.9–103.1)		224.6 (125.2–428.1)		7.3 (7.2–7.5)	
Often	53	3.8 (2.5–5.7)		32.0 (16.0–50.0)		54.0 (35.5–75.0)		67.4 (42.3–97.4)		240.6 (160.5–444.7)		7.2 (7.0–7.3)	
Going to toilet at night and interfere with sleep													
No	215	3.5 (2.5–5.0)	0.000	38.0 (23.3–48.0)	0.005	59.0 (44.0–73.0)	0.002	59.0 (36.6–93.7)	0.006	196.4 (121.6–402.8)	0.008	7.3 (7.2–7.5)	0.009
Occasionally	209	3.6 (2.5–5.0)		32.0 (20.0–49.0)		58.0 (39.9–73.1)		65.8 (40.9–109.5)		236.6 (119.7–456.1)		7.2 (7.2–7.5)	
Often	38	3.6 (2.8–5.1)		40.0 (32.0–52.0)		55.6 (47.8–67.3)		62.0 (40.9–89.9)		255.3 (170.2–333.1)		7.3 (7.2–7.6)	
Shortness of breath that interferes with sleep													
No	380	3.6 (2.5–5.0)	0.001	37.0 (24.3–49.0)	0.005	59.0 (43.9–73.2)	0.006	62.0 (38.3–98.1)	0.009	205.5 (119.6–403.4)	0.007	7.3 (7.2–7.5)	0.000
Occasionally	79	3.5 (2.3–5.2)		34.0 (19.0–50.0)		54.0 (37.0–70.0)		67.4 (36.1–128.0)		249.2 (131.0–512.1)		7.3 (7.2–7.5)	
Often	3	4.8 (2.0-.)		36.0 (11.0-)		54.0 (34.0-.)		54.4 (46.6-.)		333.8 (93.3-.)		7.4 (7.2-.)	
Coughing or snoring loudly that interferes with sleep													
No	309	3.5 (2.5–5.0)	0.008	37.0 (24.0–49.8)	0.001	58.0 (44.0–73.0)	0.000	59.5 (38.0–98.3)	0.005	201.3 (117.2–387.9)	0.008	7.2 (7.2–7.5)	0.006
Occasionally	137	4.0 (2.6–5.9)		35.0 (21.0–51.0)		59.0 (41.5–73.0)		69.7 (40.2–115.9)		243.5 (138.3–538.6)		7.3 (7.2–7.5)	
Often	16	4.2 (3.3–5.0)		36.0 (14.0–39.0)		44.5 (26.7–58.4)		61.0 (38.6–118.0)		238.1 (124.9–477.1)		7.3 (7.2–7.5)	
Feeling cold during sleeping interferes with sleep													
No	268	3.5 (2.5–5.0)	0.005	36.0 (22.0–48.0)	0.004	58.0 (41.0–73.6)	0.003	59.2 (39.0–98.7)	0.002	203.1 (120.7–388.7)	0.003	7.3 (7.2–7.5)	0.005
Occasionally	183	4.0 (2.7–5.6)		36.5 (23.0–51.3)		58.0 (42.0–73.0)		66.6 (38.3–107.4)		237.7 (127.3–475.5)		7.3 (7.2–7.5)	
Often	11	3.5 (2.0–5.0)		43.0 (36.0-)		59.0 (53.0–71.3)		65.1 (29.3–93.3)		183.5 (86.1–403.6)		7.2 (7.2–7.7)	
Feeling hot while sleeping interferes with sleep													
No	236	3.5 (2.5–5.0)	0.015	38.0 (25.0–49.0)	0.002	59.0 (42.4–73.6)	0.001	65.1 (40.9–101.0)	0.006	225.4 (126.3–425.8)	0.024	7.3 (7.2–7.5)	0.001
Occasionally	216	4.0 (2.8–5.0)		36.0 (21.3–48.8)		57.0 (42.0–71.0)		61.1 (32.9–97.2)		206.8 (116.4–413.4)		7.3 (7.2–7.5)	
Often	10	3.8 (2.5–7.1)		37.0 (16.3–61.0)		67.6 (28.8–86.9)		80.7 (43.7–123.9)		371.0 (135.5–742.4)		7.3 (7.2–7.5)	
Pain and discomfort during sleeping can interfere with sleep													
No	319	3.5 (2.5–5.0)	0.008	37.0 (25.0–49.0)	0.004	58.0 (42.9–72.0)	0.002	63.0 (38.3–97.2)	0.011	204.0 (119.3–387.8)	0.004	7.3 (7.2–7.5)	0.014
Occasionally	134	4.1 (2.7–6.0)		36.0 (20.3–51.5)		57.5 (39.7–75.0)		64.0 (38.2–121.7)		249.2 (130.1–523.0)		7.3 (7.2–7.5)	
Often	9	4.0 (2.0–5.7)		16.0 (4.0-)		59.0 (35.2–68.9)		53.3 (39.0–84.0)		183.5 (103.3–384.0)		7.3 (7.1–7.6)	
Wake up time in workday (nearly a month)													
Before 6:00	30	4.1 (3.1–6.0)	0.000	36.0 (25.5–50.0)	0.001	60.0 (35.0–73.0)	0.000	78.8 (39.1–134.0)	0.031	377.4 (151.9–564.8)	0.049	7.2 (7.2–7.5)	0.000
6:00–8:00	380	3.6 (2.5–5.0)		36.0 (22.0–48.0)		58.0 (42.0–72.8)		60.7 (38.3–96.8)		210.4 (117.4–392.8)		7.3 (7.2–7.5)	
8:00–10:00	40	3.4 (2.4–5.0)		38.0 (22.5–51.0)		54.8 (45.2–72.8)		75.2 (37.9–136.4)		235.7 (125.6–506.4)		7.5 (7.2–7.7)	
After 10:00	12	3.4 (2.2–5.8)		49.5 (34.3–57.8)		70.8 (37.0–81.2)		61.0 (38.8–121.4)		198.8 (139.9–368.0)		7.2 (7.1–7.7)	
Use of hypnotic drugs (nearly a month)													
No	452	3.6 (2.5–5.0)	− 0.101	36.0 (22.0–49.0)	0.247	58.0 (42.0–72.8)	#####	63.3 (38.3–99.5)	− 0.238	214.4 (121.8–418.6)	− 0.56	7.3 (7.2–7.5)	− 0.013
Occasionally	10	4.0 (2.5–5.8)		38.0 (14.8–51.8)		67.0 (43.9–76.5)		103.5 (34.5–171.0)		202.6 (126.0–1038.7)		7.3 (7.0–7.5)	
Feel sleepy (nearly a month)													
No	106	3.4 (2.5–5.0)	0.004	41.0 (26.5–53.0)^*^	0.016	61.5 (48.8–77.3)^*^	0.02	75.5 (43.5–120.0)	0.007	237.0 (130.3–467.9)	0.004	7.5 (7.2–7.7)^*^	0.02
Occasionally	282	3.9 (2.7–5.1)		36.0 (21.8–49.0)		57.0 (40.8–72.0)		62.1 (35.8–98.4)		218.9 (114.7–420.1)		7.3 (7.2–7.5)	
Often	74	3.5 (2.1–5.0)		34.0 (20.0–47.5)		52.7 (38.3–69.1)		59.4 (40.3–91.1)		187.0 (115.7–359.1)		7.2 (7.2–7.5)	
The length of a daily nap													
Don't nap	60	3.8 (2.5–5.3)	0.002	41.0 (13.5–48.0)	0.000	55.5 (39.1–71.0)	0.003	57.1 (35.4–104.8)	0.001	219.7 (122.0–399.4)	0.000	7.3 (7.2–7.7)^*^	0.007
Occasionally a nap	185	3.9 (2.8–5.0)		36.0 (24.0–49.0)		57.0 (41.0–72.0)		65.1 (41.0–99.0)		222.2 (128.9–449.2)		7.3 (7.2–7.5)	
Often a nap	217	3.5 (2.5–5.1)		36.0 (22.8–49.3)		59.0 (44.0–74.3)		61.8 (38.6–100.1)		204.0 (116.6–409.0)		7.2 (7.2–7.5)	
Participate in sports activities													
Never	106	4.0 (2.5–5.2)	0.004	36.5 (21.8–47.0)	0.004	54.5 (39.9–70.3)	0.002	69.1 (42.2–108.1)	0.003	255.3 (131.9–476.2)	0.010	7.3 (7.2–7.5)	0.003
Occasionally	316	3.5 (2.5–5.0)		36.5 (22.0–50.0)		59.0 (42.0–73.9)		62.8 (36.5–99.5)		200.3 (118.3–400.9)		7.2 (7.2–7.5)	
Often	40	4.0 (2.6–5.6)		31.5 (20.8–49.0)		58.7 (49.1–73.4)		54.2 (37.4–91.3)		225.4 (114.1–400.1)		7.4 (7.2–7.5)	

The value of semen volume, progressive motility, sperm concentration, sperm count, total motility, pH value represent median (25th, 75th percentiles). *P < 0.05

### Independent predictors of low semen quality by binomial logistic regression analysis

**Fig. 1:**
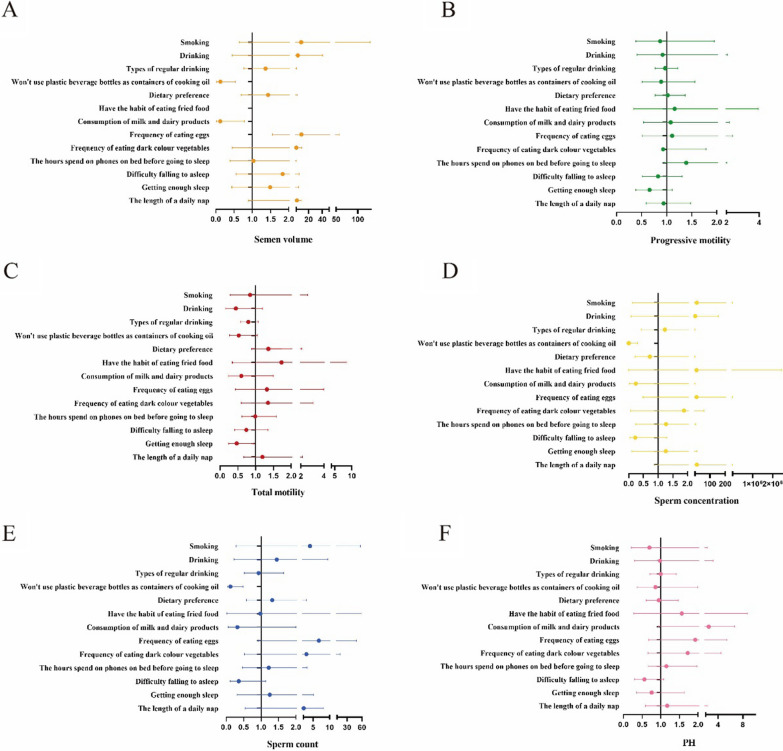
Forest plot showing the effect of different diet and lifestyle on semen volume (**A**), progressive motility (**B**), total motility (**C**), sperm concentration (**D**), sperm count (**E**), pH value (**F**). Dots represent ORs. Error bars indicate 95% CIs

Table 5 and Fig. 1 show the results of binomial logistic analysis. Abnormal semen quality parameters were defined by the guidelines of the WHO [13]. After adjusting for education state, we observed that not using plastic beverage bottles for cooking oil and condiments was a positive factor for semen volume (odds ratio [OR]: 0.12; 95% confidence interval [CI] 0.03–0.55; P = 0.006), sperm concentration (OR: 0.001; 95% CI 0.00–0.30; P = 0.012), and total sperm count (OR: 0.12; 95% CI 0.03–0.48; P = 0.003). Moreover, consuming milk and dairy products (OR: 0.11; 95% CI 0.09–0.97; P = 0.044) contributed to increased semen volume, while eggs intake may contribute to reductions in semen volume (OR: 9.41; 95% CI 1.55–57.27; P = 0.015). Finally, getting a sufficient amount of sleep was a positive factor for total sperm motility (OR: 0.47; 95% CI 0.24–0.95; P = 0.034).

**Table 5:** Binomial regression model to explore the relationship between lifestyle and dietary intake and semen quality

Characteristic	Semen volume			Total motility			Progressive motility			Sperm concentration			Sperm count			pH value		
	(< 1.5 ml vs ≥ 1.5 ml)			(< 40% vs ≥ 40%)			(< 32% vs ≥ 32%)			(< 15 × 10^6^/ml vs ≥ 15 × 10^6^/ml)			(< 39 × 10^6^ vs ≥ 39 × 10^6^)			(< 7.2vs ≥ 7.2)		
	OR(95%CI)	*P*	*R*^*2*^	OR(95%CI)	*P*	*R*^*2*^	OR(95%CI)	*P*	*R*^*2*^	OR(95%CI)	*P*	*R*^*2*^	OR(95%CI)	*P*	*R*^*2*^	OR(95%CI)	*P*	*R*^*2*^
Smoking	9.06 (0.64–128.28)	0.103	0.326	0.85 (0.28–2.60)	0.771	0.208	0.87 (0.39–1.95)	0.735	0.049	14.44 (0.13–1619.17)	0.267	0.561	4.04 (0.29–56.36)	0.300	0.313	0.69 (0.21–2.32)	0.55	0.128
Drinking	4.27 (0.45–40.73)	0.207		0.45 (0.17–1.20)	0.111		0.92 (0.41–2.05)	0.830		3.46 (0.08–155.40)	0.522		1.45 (0.22–9.49)	0.701		0.98 (0.30–3.21)	0.968	
Types of regular drinking	1.36 (0.77–2.41)	0.294		0.80 (0.59–1.08)	0.146		0.97 (0.77–1.22)	0.792		1.23 (0.43–3.47)	0.698		0.93 (0.52–1.65)	0.793		1.00 (0.71–1.41)	0.993	
Won’t use plastic beverage bottles as containers	0.12 (0.03–0.55)	0.006^*^		0.53 (0.27–1.05)	0.067		0.89 (0.51–1.56)	0.685		0.00 (0.00–0.30)	0.012^*^		0.12 (0.03–0.48)	0.003^*^		0.86 (0.37–1.99)	0.721	
Dietary preference	1.43 (0.69–2.97)	0.34		1.36 (0.89–2.09)	0.155		1.02 (0.77–1.37)	0.874		0.72 (0.22–2.35)	0.587		1.32 (0.58–2.98)	0.51		0.95 (0.61–1.47)	0.819	
Have the habit of eating fried food	0.04 (0.00–1.30)	0.071		1.73 (0.35–8.46)	0.5		1.16 (0.34–3.94)	0.818		12.42 (0.00–2434424.12)	0.685		0.98 (0.02–58.68)	0.994		1.55 (0.28–8.77)	0.618	
Consumption of milk and dairy products	0.11 (0.02–0.83)	0.032^*^		0.60 (0.24–1.50)	0.274		1.08 (0.54–2.17)	0.819		0.24 (0.02–3.86)	0.315		0.32 (0.05–2.00)	0.221		2.50 (0.93–6.70)	0.069	
Frequency of eating eggs	9.41 (1.55–57.27)	0.015^*^		1.32 (0.43–3.99)	0.626		1.11 (0.52–2.37)	0.789		12.37 (0.49–311.32)	0.126		6.70 (0.92–49.07)	0.061		1.92 (0.68–5.44)	0.219	
Frequency of eating dark colour vegetables such as yellow, red and purple	2.10 (0.45–9.76)	0.342		1.35 (0.59–3.07)	0.477		0.93 (0.48–1.79)	0.822		1.88 (0.06–61.60)	0.723		2.91 (0.53–15.93)	0.219		1.72 (0.66–4.50)	0.272	
The average number of hours spent on phones on bed before going to sleep each day	1.03 (0.39–2.74)	0.947		0.99 (0.62–1.58)	0.965		1.39 (0.95–2.02)	0.088		1.27 (0.24–6.75)	0.78		1.22 (0.47–3.18)	0.681		1.15 (0.67–1.97)	0.606	
Difficulty falling asleep	1.83 (0.56–5.99)	0.318		0.74 (0.41–1.35)	0.329		0.83 (0.52–1.31)	0.417		0.22 (0.04–1.29)	0.093		0.36 (0.11–1.13)	0.079		0.56 (0.29–1.08)	0.082	
Getting enough sleep	1.48 (0.44–5.06)	0.529		0.47 (0.24–0.95)	0.034^*^		0.66 (0.39–1.11)	0.116		1.26 (0.11–14.29)	0.855		1.25 (0.31–5.06)	0.753		0.76 (0.35–1.63)	0.477	
The length of a daily nap	2.93 (0.89–9.62)	0.077		1.19 (0.66–2.14)	0.572		0.94 (0.59–1.48)	0.774		14.95 (0.89–250.03)	0.06		2.11 (0.55–8.15)	0.279		1.17 (0.59–2.32)	0.664	

^*^P < 0.05

## Discussion

In this cross-sectional study, we enrolled 466 couples who were attempting to conceive, and focused on the dietary and lifestyle factors that affect the fertility of male partners. Several independent factors have been found to correlate with semen quality, and some of those identified in the current study are supported by previous studies. Indeed, smoking and alcohol consumption have been associated with reduced sperm motility and are well-known factors that affect semen quality. A new systematic evaluation and meta-analysis of 5,865 men showed that smoking was associated with reduced sperm count and viability, with a more pronounced deterioration in semen quality observed in moderate and heavy smokers [24]. The effect of smoking on spermatogenesis may be explained by dual mechanisms. First, a reduction in T concentration in the testicular tissue due to impaired Leydig cell function may result in disturbed spermatogenesis, spermiogenesis, and epididymal function, which may explain the disturbances in sperm motility and morphological characteristics. Second, nicotine or catecholamines released during smoking can directly affect steroidogenesis and spermatogenesis [25]. In conclusion, smoking in men may affect their fertility by interfering with normal testicular steroid production and spermatogenesis due to stress-induced overactivity of the adrenal medulla and adrenal cortex. Furthermore, the association between chronic alcohol consumption and poor semen quality is mainly due to the development of oxidative stress and its genotoxic effects on hormonal regulation and DNA integrity, which in turn affect the health of the offspring [17]. Sleep quality is another factor that has been widely reported to be associated with semen quality, and has also been found to affect sperm motility [26]. Indeed, Chen et al. assessed the relationship between sleep quality and semen parameters in 842 healthy men, and found that poor sleep quality was associated with impaired semen parameters [27]. Moreover, in 2013, Jensen et al. reported an inverse relationship between sleep disturbance and sperm concentration, total count, and percentage of normal morphology in 953 healthy Danish men. These findings are consistent with our results [28]. Although living habits, such as diet preference and using plastic beverage bottles as containers for cooking oil, have been seldom reported by other researchers, we found close associations between them and semen quality. Moreover, improper spice containers can cause harmful substances in the plastic to leach into the spice, which can be absorbed by the body and lead to a reduction in semen quality, and even birth defects in offspring [29]. Indeed, a recent study by Xia indicated that microplastics (MP) have reproductive toxicity and transgenerational effects in aquatic species, with potential adverse effects on mammalian reproduction [30]. Besides, Jin et al. demonstrated that long-term exposure to PS-MPs at concentrations equivalent to environmental contamination resulted in impaired testicular tissue structure, reduced sperm quality, and decreased testosterone levels, leading to male reproductive toxicity in mice. Among them, the PS-MPs-induced decrease in testosterone levels was achieved through inhibition of the LH-mediated LHR/cAMP/PKA/StAR pathway [31]. Our results also suggested that food choice is vital to semen quality, with foods such as eggs and roughage being associated with changes in semen quality. However, these food preference factors and their association with semen quality have been rarely reported. We also noticed that the consumption of milk wasbeneficial to the total sperm motility and concentration; however, the classification of dairy products was unclear owing to the small sample size. The literature on the relationship between dairy products is inconclusive. Although some studies have suggested that dairy products may be a risk factor for poor semen parameters, others do not support this theory. In a case–control study comparing the dietary habits of men with oligozoospermia and normospermia, case subjects consumed higher amounts of whole milk products (yogurt, whole milk, cheese, and semi-fermented milk) and lower amounts of skim milk than control subjects [32]. Moreover, in an American cohort study, intake of low-fat dairy products was associated with higher sperm concentration and better motility [33]. Furthermore, in a study of young men engaged in physical labor, the intake of full-fat dairy products, especially cheese, was adversely associated with normal sperm morphological characteristics and progressive sperm motility [34]. However, in another study of men in a Dutch hospital, dairy intake was not associated with semen quality [35]. While most studies support the benefits of low-fat versus the harmful effects of full-fat dairy products, more studies, especially randomized trials, are needed to draw well-supported conclusions.

Semen quality is easily influenced by one’s own behavior and the environment [36, 37]. However, many factors cannot be studied and discussed simultaneously. By sorting the factors by group, we have the opportunity to analyze the effects of interrelated factors on semen quality and, in the future, it will be possible to combine different groups of factors before applying medical data, artificial intelligence, and machine learning to construct a mathematical model to evaluate male fertility. The results of this intuitive evaluation will assist doctors in pre-pregnancy clinics with selecting an appropriate solution for each case, and provide patients with a set of guidelines to follow to reduce their exposure to risk factors and consequently, restore their fertility. Avoiding exposure to high-risk factors before pregnancy will save couples preparing for pregnancy from expensive medical costs and provide a scientific basis for precise fertility interventions.

Originally, assisted reproductive technology (ART) was intended to help couples with organic diseases become pregnant. Since its first application, more than 300,000 infants have been born in China as a result of ART [38]. However, ART is frequently believed to be abused, with excessive medical treatments [39–41]. It has also been reported that ART may lead to higher risks of gestational diseases, hypertension, and other pregnancy-related diseases. Even after controlling for known risk factors, such as maternal age, weight, and poor lifestyle habits, ART is associated with a higher risk of adverse perinatal outcomes, such as placenta previa, premature abruption, antepartum hemorrhage, low amniotic fluid, cesarean delivery, preterm delivery, very low birth weight, low birth weight, and increased risk of perinatal mortality [42–45]. Therefore, if couples can successfully become pregnant naturally by avoiding exposure to risk factors, infertility treatments may be reserved for couples with the greatest need, preventing excessive application of ART and avoiding unnecessary ART expenses.

To achieve our expectations, we must recruit more couples with infertility concerns and expand the sample size for a more reliable result. In the meantime, to improve the accuracy of our results, we plan to modify our questionnaires according to the reflections of our patients.

## Conclusions

Overall, our results demonstrated that drinking, smoking, using plastic bottles for condiment containers, dietary preference, sleep, and consumption of milk, egg, and roughage are related to semen quality. However, as our cohort was comparatively small, we plan to increase our sample size to verify our results. Additionally, the specific mechanisms by which risk factors, such as high fat, red meat, processed meat, refined grains, candy and sweet drinks, unhealthy eating patterns, and long periods of sedentary work condition, affect semen quality are still unknown and require further research [46].

## Data Availability

All data generated or analyzed during this study are included in this published article.

## Abbreviations

CASA: Computer-Aided Sperm Analysis
CI: Confidence Interval
OR: Odds Ratio
SD: Standard Deviation
WHO: World Health Organization
